# Sorafenib improves rituximab and ofatumumab efficacy by decreasing the expression of complement regulatory proteins

**DOI:** 10.1038/bcj.2015.27

**Published:** 2015-04-10

**Authors:** M Dwojak, M Bobrowicz, J Bil, K Bojarczuk, B Pyrzynska, M Siernicka, A Malenda, E Lech-Maranda, W Tomczak, K Giannopoulos, J Golab, M Winiarska

**Affiliations:** 1 Department of Immunology, Centre for Biostructure Research, Medical University of Warsaw, Warsaw, Poland; 2 Postgraduate School of Molecular Medicine, Warsaw, Poland; 3 Department of Invasive Cardiology, Central Clinical Hospital of the Ministry of Interior, Warsaw, Poland; 4 Department of Haematology, Institute of Haematology and Transfusion Medicine, Warsaw, Poland; 5 Department of Haematology and Transfusion Medicine, Centre of Postgraduate Medical Education, Warsaw, Poland; 6 Department of Haematooncology and Bone Marrow Transplantation, Medical University of Lublin, Lublin, Poland; 7 Department of Experimental Haematooncology, Medical University of Lublin, Lublin, Poland; 8 Institute of Physical Chemistry, Polish Academy of Sciences, Department 3, Warsaw, Poland

Despite major therapeutic advances in chronic lymphocytic leukaemia (CLL), there is still no curative strategy and new treatment approaches with improved efficacy are urgently needed. Although showing clinical activity in CLL patients, anti-CD20 monoclonal antibodies in chemoimmunotherapeutic regimens display rather limited efficacy. Thus combinations of targeted therapies with immunotherapeutic approaches might constitute a promising strategy to enhance antitumour effects. To date, several preclinical studies have reported the sensitivity of CLL cells to sorafenib, an oral compound targeting the activity of numerous membrane-bound and cytosolic kinases (BRAF, c-Raf, vascular endothelial growth factor receptor, platelet-derived growth factor receptor, fms-related tyrosine kinase 3, c-Kit), suggesting that it could represent a viable option as a therapy for CLL.^1, 2^ Sorafenib has been reported to induce proliferation arrest and apoptosis in B-cell tumours. Antitumour effects of sorafenib in CLL are not abrogated by pro-survival signals from stromal cells indicating that treatment with sorafenib may overcome drug resistance.^1, 2, 3^

At present, sorafenib is being investigated in >500 clinical trials, mostly in solid tumours and also in B-cell malignancies. Recently, the combination of perifosine (AKT/phosphoinositide 3-kinase inhibitor) and sorafenib has demonstrated promising activity in Hodgkin's lymphoma patients. Phase II studies in diffuse-large B-cell lymphoma^4^ and refractory B-cell lymphoma patients^5^ revealed good tolerance to sorafenib. Moreover, a phase I/II clinical trial is currently initiated to assess the effects of sorafenib in CLL patients.

Considering the accumulating number of preclinical and clinical studies confirming that combined regimens including sorafenib might be beneficial in CLL therapy, in the present study we assess the influence of sorafenib on antitumour activity of anti-CD20 monoclonal antibodies (mAbs) in lymphoma/leukaemia cell lines and primary CLL samples. We observe that sorafenib potentiates antitumour effects of anti-CD20 mAbs, an effect likely mediated by downregulation of membrane-bound complement regulatory proteins (mCRPs).

In our primary experiments using a standard MTT (3-(4,5)-dimethylthiazol-2-yl-2,5-diphenyltetrazolium bromide) cytotoxicity assay, we observe that a 48-h preincubation of Raji, Ramos or Daudi cells (Burkitt's lymphomas), as well as DoHH2 cells (follicular lymphoma), with sorafenib significantly potentiates the ability of rituximab to induce complement-dependent cytotoxicity (CDC; Figure 1a). Also, flow cytometry experiments with antibodies recognizing neo-epitope within C5b-9 reveal strong augmentation in incorporated membrane attack complexes (MAC) in Raji cells preincubated with sorafenib (Figure 1b, Supplementary Figure 1A). In rituximab-resistant CLL cells (MEC-1), sorafenib fails to increase rituximab-mediated CDC but effectively potentiates CDC triggered by ofatumumab (Figure 1c), another anti-CD20 mAb reported to strongly induce CDC.

As anti-CD20 mAbs also activate antibody-dependent cell-mediated cytotoxicity (ADCC), we sought to determine the influence of sorafenib on natural killer (NK) cells. The results of preclinical studies reporting immunoregulatory effects of sorafenib on NK cells activity are controversial and not conclusive. For example, sorafenib was shown to alter the microenvironment of hepatocellular carcinomas and trigger activation of NK cells by restoring classical macrophage polarization.^6^ Moreover, sorafenib sensitized hepatocellular carcinoma cells to NK cell-mediated functions by inhibiting the shedding of major histocompatibility class I-related chain A ectodomain.^7^ When combined with human anti-glypican 3 antibody, sorafenib showed additive effect on ADCC in a xenograft model of human liver cancer *in vivo*.^8^ However, sorafenib has also been reported to have a suppressive effect on NK cells acting directly on NK cell proliferation and function by inhibition of phosphoinositide 3-kinase and extracellular signal-regulated kinase signalling pathways.^9, 10^ In line with these latter reports, we observe a suppression of NK cell function in ADCC assay measuring CD107a mobilization (Figure 1d, left panel) in a model where both NK cells and target cells are incubated for 4 h with rituximab in the presence of sorafenib. However, we also employed a clinically more relevant model, where both target Raji cells and effector NK cells are preincubated with sorafenib for 48 h and further co-incubated for 4 h with rituximab in the presence of sorafenib. In this model, sorafenib has no influence on rituximab-mediated ADCC (Figure 1d, right panel) indicating the possibility that sorafenib inhibits the activity of NK cells, and it simultaneously sensitizes target tumour cells to NK cell-mediated killing. Further studies are clearly needed to determine in more detail the impact of sorafenib on the complex interplay between target cancer cells and effector NK cells.

To further determine the mechanisms of potentiated CDC, we measured the expression levels of CD20 as well as other B-cell membrane proteins known to regulate CDC. Flow cytometry reveals a significant increase of surface CD20 levels in Raji (Figure 1e) and MEC-1 cells (Figure 1f), as well as an increased binding of rituximab to Raji cells (data not shown). Simultaneously, we observe that sorafenib in a dose-dependent manner significantly decreases surface levels of mCRPs, including CD46 (Figures 1e and f), CD55 (Figure 1e) and CD59 (Figure 1f). Also, in a set of 22 primary tumour samples preincubated with sorafenib for 48 h we observe significantly decreased surface levels of CD46, CD55 and CD59 (Figure 2a, Supplementary Figure S2A). At the same time, the levels of surface CD20, as well as of other antigens such as CD19, CD37 and CD38, remain roughly unchanged in primary CLL cells incubated with sorafenib (Supplementary Figure S2B). In nine consecutive primary CLL samples, sorafenib significantly potentiates O-CDC (at doses 1 and 10 μg/ml) (Figure 2b). Collectively, we demonstrate that sorafenib significantly downregulates mCRPs and this effect is sufficient to sensitize tumour cells to anti-CD20 mAbs-mediated CDC.

Sorafenib has been reported to inhibit phosphorylation of signal transducer and activator of transcription 3 (STAT3) and to block STAT3 signalling in several tumour cell lines. Some *in vitro* studies suggest that constitutive phosphorylation of STAT3 is a hallmark of CLL and can be considered as a therapeutic target in this disease.^11^ Sorafenib targets Raf-1 and receptor tyrosine kinases, including vascular endothelial growth factor receptor, platelet-derived growth factor receptor and c-Kit, and inhibits STAT3 activity as a point of convergence for these tyrosine kinases. Consistently with these findings, we observe a dose-dependent inhibition of STAT3 phosphorylation (Tyr 705) in Raji cells incubated with sorafenib for 48 h (Supplementary Figure S3A). Noteworthy, CD46 promoter contains the STAT3 consensus-binding site,^12^ and both CD46 and CD55 promoters were found to bind STAT3 transcription factor.^13^ We also analysed the promoters of CD55 and CD59 and found several putative STAT3-binding sites (Supplementary Figure S4). Somewhat surprisingly, a transcriptional analysis of cells reveals that mRNA levels of mCRPs in Raji cells (Supplementary Figure S3B) and primary CLL cells (Supplementary Figure S3C) do not change upon incubation with sorafenib as measured with quantitative reverse transcriptase-PCR. It is possible that STAT3-mediated effects induced by sorafenib are carried out by other nontranscriptional mechanisms. It has been already shown that independently of transcriptional activity STAT3 can serve as a protein scaffold to facilitate the interaction between phosphatidylinositol 3-kinase and the type I interferon receptor.^14^

To further determine whether downregulation of mCRPs is attributed to pSTAT3 inhibition, we assessed the effects of SC-1, a novel sorafenib analogue devoid of Raf-1 but retaining potent STAT3 inhibitory activity, on mCRPs levels. SC-1 dose-dependently downregulates CD46, CD55 and CD59 levels in MEC-1 cells (Figure 2c). Importantly, mCRPs downregulation corresponds with sensitization of MEC-1 cells to both R-CDC and O-CDC (Figure 2d). Collectively, our results indicate that STAT3 may potentially have a role in the regulation of mCRPs' levels. However, further studies are required to fully elucidate the mechanisms of this phenomenon.

Activation of classical complement cascade has a role in the therapeutic efficacy of several unconjugated therapeutic antibodies. For example, in unmanipulated whole blood assays the activity of ofatumumab has been shown to be fully complement-dependent and further increased by blocking CD55 and CD59.^15^ Induction of CDC is also important for alemtuzumab action. Moreover, while trastuzumab alone does not induce CDC, in combination with pertuzumab it kills tumour cells in a complement-dependent manner. Although tumour cells can overexpress mCRPs, neutralization or downregulation of complement inhibitors has been proposed to increase mAb-induced CDC. Here we add another potential therapeutic approach—a drug-mediated downregulation of mCRPs levels that seems to be of clinical significance.

In many *in vitro* studies sorafenib as a single-agent displays a strong pro-apoptotic activity in CLL cells, even in the presence of pro-survival factors derived from stromal cells. Our *in vitro* studies, where sorafenib shows potentiated antitumour effects when combined with anti-CD20 mAbs, indicate that sorafenib constitutes a promising strategy for sensitization of tumour cells to mAb-mediated CDC and support its use as a new therapeutic option in CLL.

## Figures and Tables

**Figure 1:**
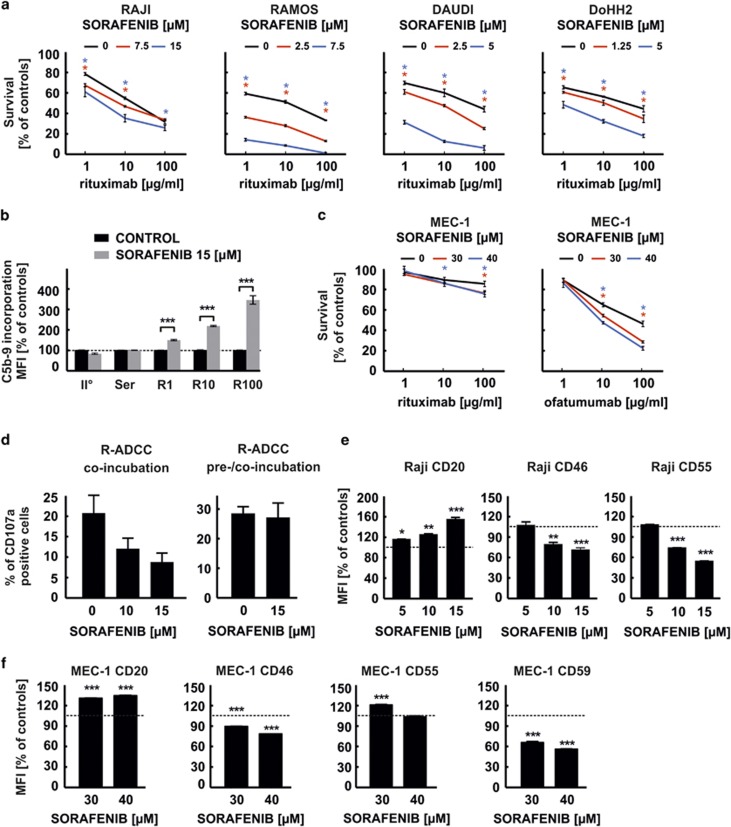
Sorafenib potentiates R-CDC and decreases surface levels of mCRPs in various lymphoma/leukaemia cell lines. (**a**) In the CDC assay, 100 000 cells (control or pretreated with sorafenib for 48 h) were incubated with rituximab (1–100 μg/ml) and 10% human plasma as a source of complement for 60 min at 37 °C in fetal bovine serum (FBS)-free RPMI medium. After incubation, MTT reduction assay was performed as described elsewhere.^16^ GraphPad Software (La Jolla, CA, USA) was used to determine statistical significance with Mann–Whitnney *U* test, *P<0.05 vs control. (**b**) 500 000 cells (control or pretreated with sorafenib for 48 h) were incubated for 30 min in FBS-free medium at 37 °C with increasing concentrations of rituximab and 10% human plasma. After incubation, cells were washed with ice-cold phosphate-buffered saline (PBS), centrifuged and stained with anti-MAC primary antibody (mouse monoclonal, clone aE11, Dako (Glostrup, Denmark), final concentration 250 ng/test). After 30-min incubation on ice, cells were washed and incubated for 30 min at room temperature (RT) in the dark with secondary antibody (donkey, anti-mouse-IgG-Alexa488-conjugated, 1:100, Life Technologies, Waltham, MA, USA). Cells were analysed on a FACScan (Becton, Dickinson and Company, Franklin Lakes, NJ, USA) using the CellQuest Pro Software Version 5.2 (BD Biosciences, San Jose, CA, USA). Statistical significance was determined with two-way analysis of variance (ANOVA) test with Bonferroni's correction, ***P<0.001 vs control. (**c**) CDC assay with rituximab or ofatumumab using MEC-1 cells was performed as described above. Statistical significance was determined with Mann–Whitnney *U* test, *P<0.05 vs control. (**d**) For the degranulation assay, natural killer (NK) cells were negatively isolated from peripheral blood mononuclear cells of healthy donors using the EasySep Human NK cell Enrichment Kit (STEMCELL Technologies, Vancouver, BC, Canada) and stimulated overnight with human recombinant interleukin-2 (Proleukin, 100 IU/ml; Chiron, Barcelona, Spain) and interferon-α (Roferon-A, 100 IU/ml; F. Hoffmann-La Roche AG, Basel, Switzerland). NK effector cells were incubated with target Raji cells (at E:T ratio 1:1) and rituximab (100 μg/ml) in the presence of anti-CD107a fluorescein isothiocyanate (FITC)-conjugated antibody (BD Biosciences), GolgiStop (BD Biosciences) and sorafenib for 4 h (left and right panels). Additionally, in preincubation/co-incubation assay (right panel) both Raji and NK effector cells were preincubated with 15 μM sorafenib for 24 h. Upon incubation, cells were stained with PE-Vio770-conjugated anti-CD56 (Miltenyi Biotec GmbH, Bergisch Gladbach, Germany), PerCP-Cy5.5-conjugated anti-CD3 (BD Biosciences) and Fixable Viability Dye (eBioscience, San Diego, CA, USA). The percentage of CD107a-positive NK cells was evaluated with FACSAria III using the BD FACSDiva Software v 6.1.3. (**e** and **f**) To assess the surface level of membrane antigens, Raji (**e**) or MEC-1 (**f**) cells were incubated with fluorochrome-conjugated primary antibody for 30 min at RT in the dark. The following FITC-conjugated antibodies were used (all from Becton, Dickinson and Company): IgG1 (isotypic control, clone X40), anti-CD20 (clone L27), anti-CD46 (clone E4.3), anti-CD55 (clone IA10); and anti-CD59 (clone H19 ). After incubation, cells were rinsed with PBS twice and re-suspended in PBS supplemented with propidium iodide (PI) at a final concentration of 4 μg/ml. The mean fluorescence intensity (MFI) of PI-negative cells served as determinant of antigen's expression level. Cells were analysed on a FACScan as described above. Statistical significance was determined with one-way ANOVA test with Tukey's correction, ***P<0.001, **P<0.01, *P<0.05 vs control.

**Figure 2:**
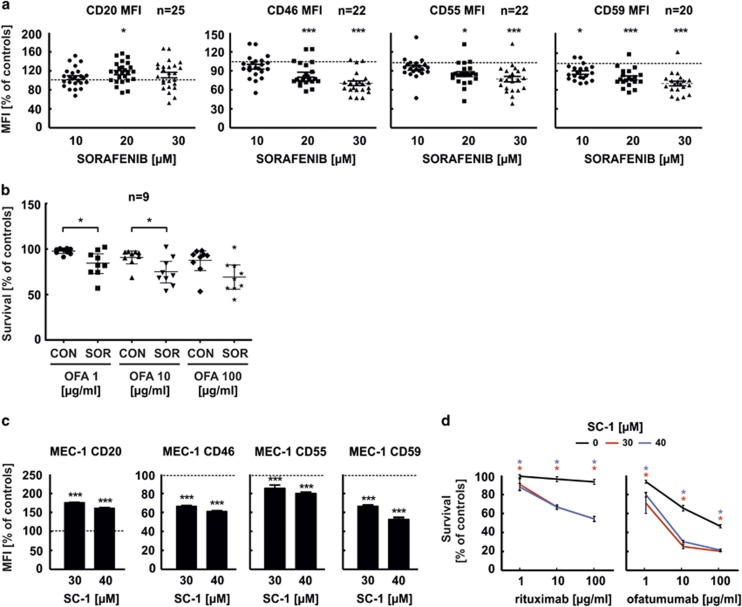
Sorafenib potentiates O-CDC and decreases surface levels of mCRPs in primary CLL cells. SC-1, a sorafenib analogue, downregulates CRPs and potentiates R-CDC and O-CDC in MEC-1 cell line. (**a**) Peripheral blood mononuclear cells from patients suffering from CLL were isolated from full blood using Histopaque-1077 (Sigma Aldrich, St Louis, MA, USA). Approval for the study was obtained from the Institutional Review Board of the Medical University of Warsaw and was conducted according to the Declaration of Helsinki. Each patient signed an informed consent for the procedures. Primary cells were treated with increasing concentrations of sorafenib for 48 h. Flow cytometry staining was performed as described above. Statistical significance was determined with one-way analysis of variance (ANOVA) test with Tukey's correction, ***P<0.001, *P<0.05 vs control. (**b**) CLL primary cells preincubated with 20 μM sorafenib for 48 h were washed and incubated with ofatumumab and 10% human plasma for 4 h. Cell viability in CDC assay was measured with propidium iodide staining using FACScan. Statistical significance was determined with Mann–Whitnney *U* test, *P<0.05 vs control. (**c**) Flow cytometry staining of surface antigens of MEC-1 cell line preincubated for 48 h with increasing concentrations of SC-1 was performed as described above. Statistical significance was determined with one-way ANOVA test with Tukey's correction, ***P<0.001 vs control. (**d**) CDC assay was performed with MEC-1 cells preincubated with SC-1 for 48 h as described above. Statistical significance was determined with Mann–Whitnney *U* test, *P<0.05 vs control.
