# Supplementary material for: Sorafenib improves rituximab and ofatumumab efficacy by decreasing the expression of complement regulatory proteins
Source: Blood Cancer J. 2015 Apr 10;5(4):e300–. doi: 10.1038/bcj.2015.27 (PMC4450327; doi:10.1038/bcj.2015.27)
Supplement: Supplementary Figure Legends [file bcj201527x1.docx]

**Supplemental Figure 1. Sorafenib augments incorporation of MAC**

Representative histograms with MFI values from MAC incorporation assay with Raji cells as described in the Fig. 1B.

**Supplemental Figure 2. Influence of sorafenib on surface levels of complement regulating proteins and other antigens in CLL primary cells.**

A-B. CLL primary cells from 3 selected donors were pre-incubated for 48h with sorafenib. To assess the surface level of membrane antigens cells were incubated with fluorochrome-conjugated primary antibody for 30 min at RT in the dark. The following FITC-conjugated antibodies were used (all from Becton Dickinson): IgG1 (isotypic control, clone X40), anti-CD20 (clone L27), anti-CD46 (clone E4.3), anti-CD55 (clone IA10); anti CD59 (clone H19), anti-CD19 (clone HIB19), anti-CD37 (clone M-B371), anti-CD38 (clone HIT2). After incubation cells were rinsed with PBS twice and re-suspended in PBS supplemented with propidium iodide at final concentration 4 μg/mL. The mean fluorescence intensity (MFI) of PI-negative cells served as determinant of antigen’s expression level. Cells were analyzed on a FACScan as described before. Prism software (GraphPad) was used to determine statistical significance with unpaired t-test, *** P < 0.001, ** P < 0.01, * P < 0.05.

**Supplemental Figure 3. Influence of sorafenib on STAT3 phosphorylation and mRNA levels of complement regulating proteins.**

1. For Western blotting lysates from Raji cells pre-incubated with increasing concentrations of sorafenib were prepared as described elsewhere (ref). Protein concentration was measured using Bio-Rad Protein Assay. 15μg of whole-cell proteins were separated by SDS-PAGE and transferred onto Protran nitrocellulose membranes (Schleicher & Schuell BioScience). Membranes were blocked with non-fat dry milk or bovine serum albumin (both 5% w/v in TBST) and incubated overnight with following primary antibodies (all in dilution 1:1000): rabbit monoclonal anti-Stat3 (clone 79D7, Cell Signaling), rabbit monoclonal anti-Phospho-Stat3 (clone D3A7, Cell Signaling), mouse monoclonal anti-α-tubulin (clone DM1A, Calbiochem). After extensive washing with TBST membranes were incubated for 1h with corresponding HRP-coupled secondary antibodies from Jackson Immuno Research (in dilution 1:10 000). The chemiluminescence reaction for HRP was developed using custom-made chemiluminescence reagent (described in ref JBC) and visualized with Stella 8300 bioimager (Raytest).
2. For quantitative Real-Time PCR assay universal RNA Purification Kit (EURx) was used to extract total RNA from Raji cells pre-incubated with sorafenib for 48h. RNA was spectrophotometrically quantified and equal amounts were primed with oligodT for following cDNA synthesis performed with AMV reverse transcriptase (EURx). Obtained cDNA served as a matrix in the subsequent quantitative Real-Time PCR reaction performed using LightCycler® Fast Start DNA Master PLUS SYBRGreen I and LightCycler 480 II device (both from Roche) according to the manufacturer’s recommendations. The following specific primers were used for the following genes of target: *CD46*, *CD55*, *CD59,* or reference genes: *ACTB*, *RPL29.*
3. qRT-PCR experiment with cDNA obtained from cells of 3 donors was performed as described above.

**Supplemental Figure 4. Analysis of consensus binding sites for STAT3 transcription factor in CD46, CD55 and CD59 promoters.**
