# Supplementary figures and images for: Sorafenib improves rituximab and ofatumumab efficacy by decreasing the expression of complement regulatory proteins
Source: Blood Cancer J. 2015 Apr 10;5(4):e300–. doi: 10.1038/bcj.2015.27 (PMC4450327; doi:10.1038/bcj.2015.27)

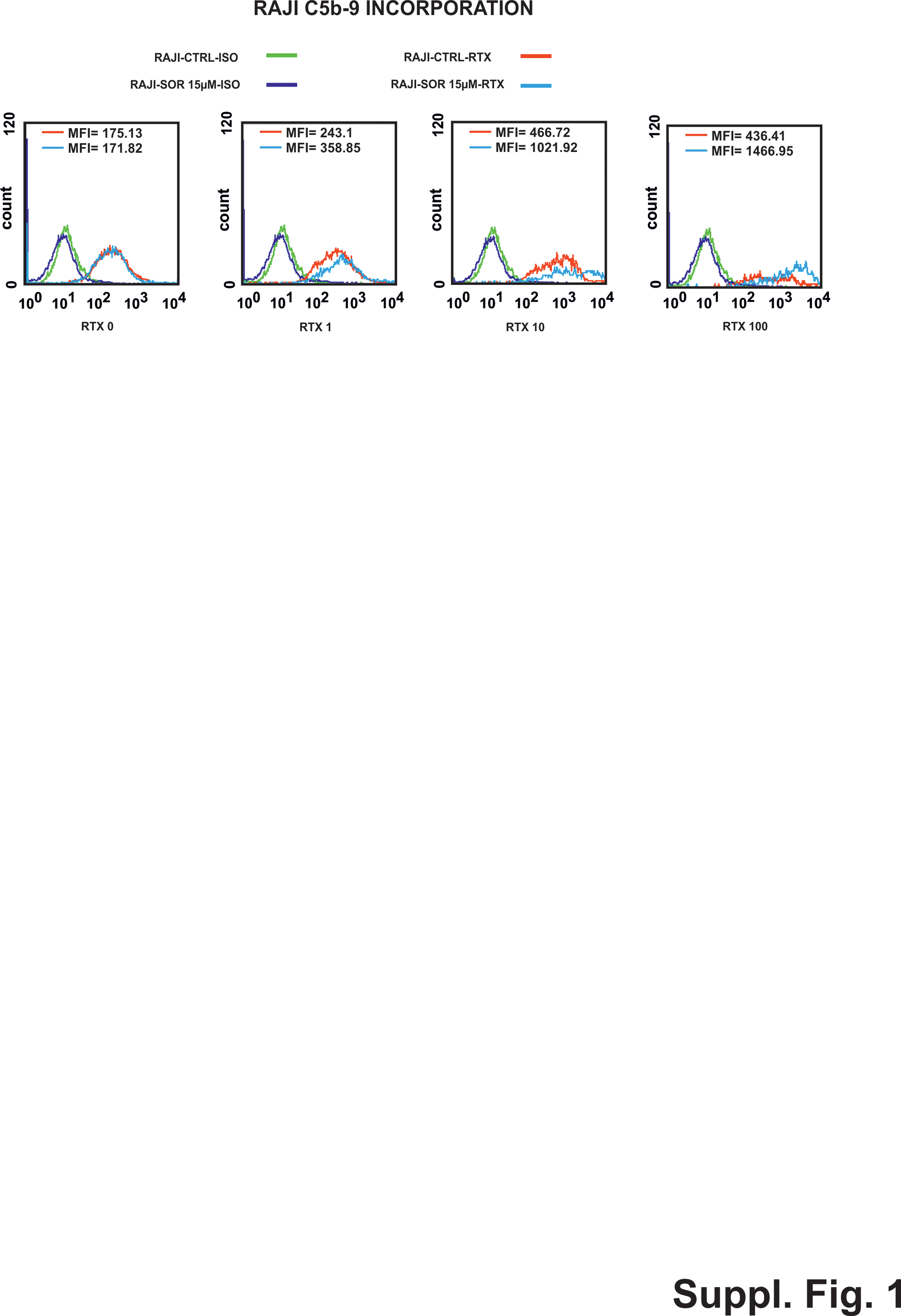

Supplement: Supplementary Figure 1 [file bcj201527x2.tif]

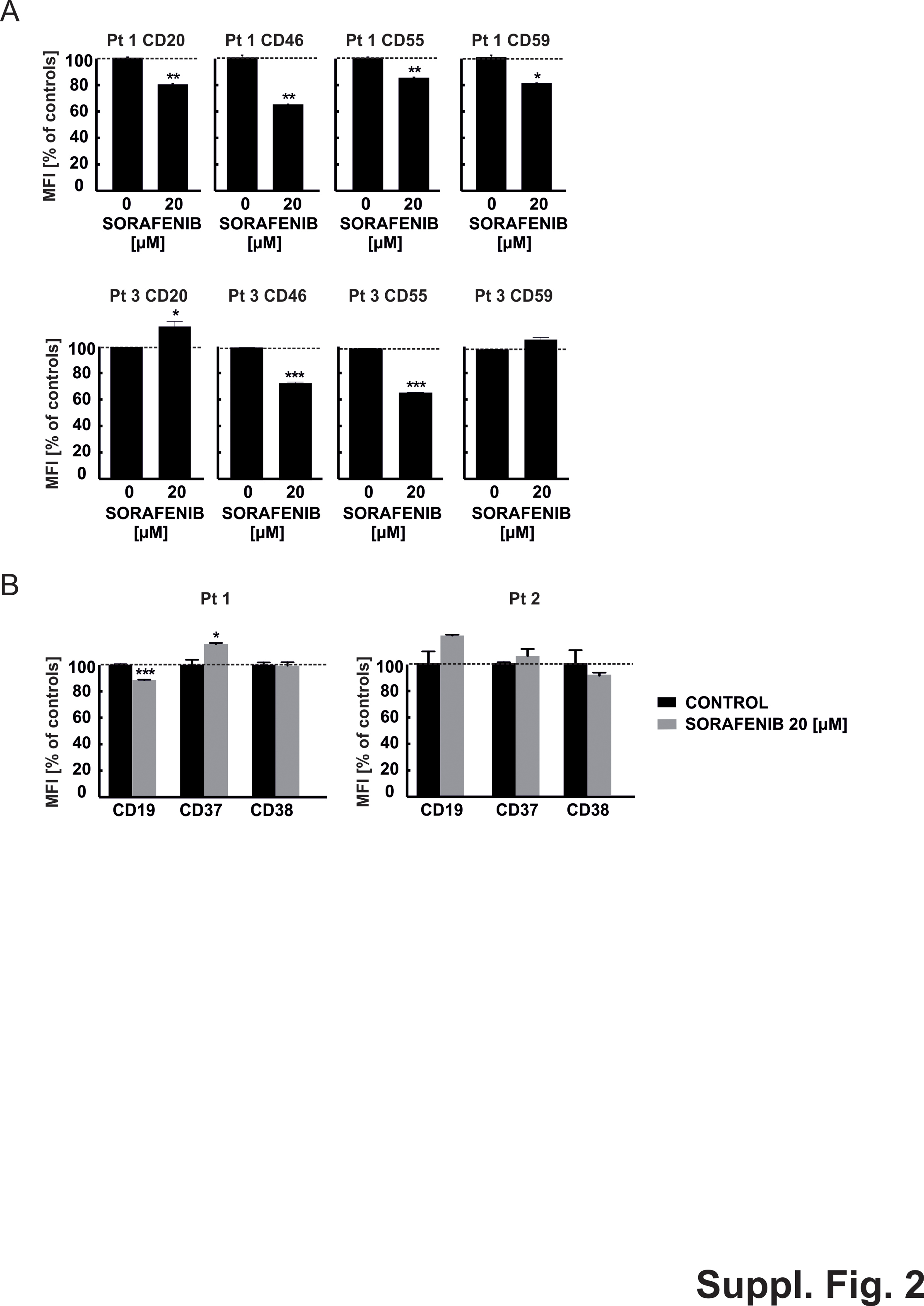

Supplement: Supplementary Figure 2 [file bcj201527x3.tif]

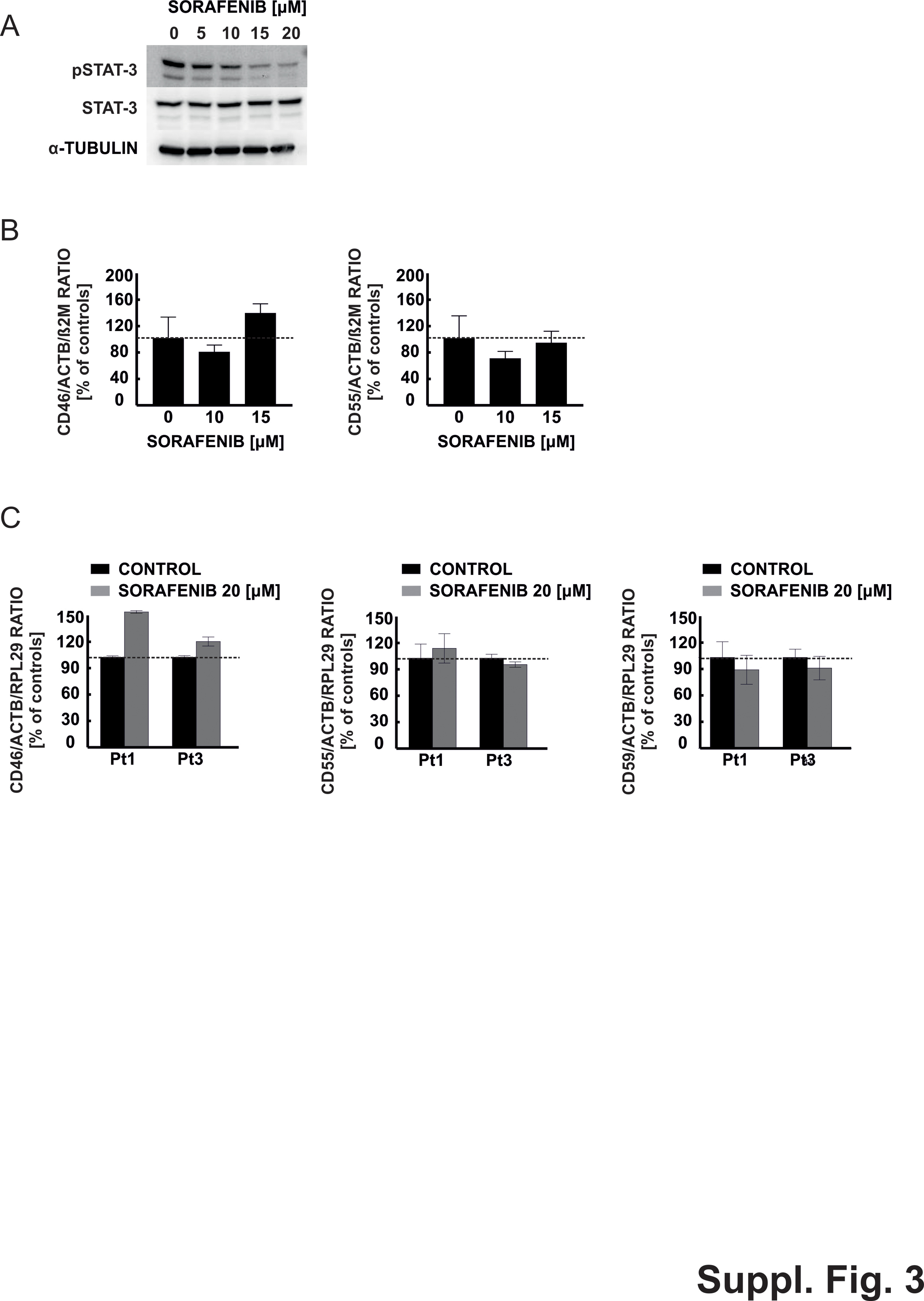

Supplement: Supplementary Figure 3 [file bcj201527x4.tif]

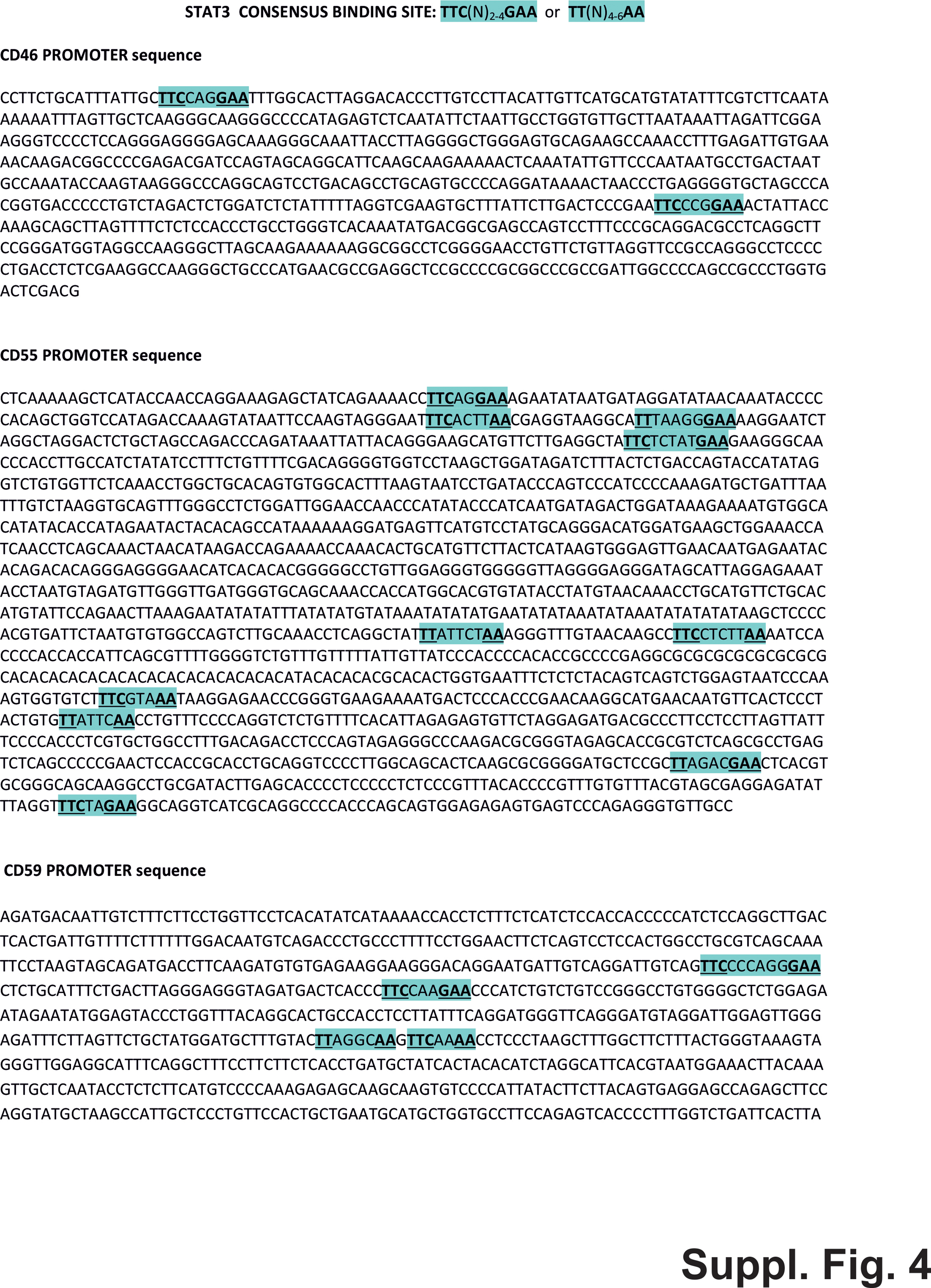

Supplement: Supplementary Figure 4 [file bcj201527x5.tif]
